# Asthma caused by occupational exposures is common – A systematic analysis of estimates of the population-attributable fraction

**DOI:** 10.1186/1471-2466-9-7

**Published:** 2009-01-29

**Authors:** Kjell Torén, Paul D Blanc

**Affiliations:** 1 Department of Occupational and Environmental Medicine, Sahlgrenska University Hospital, Göteborg, Sweden; 2 Division of Occupational and Environmental Medicine, University of California San Francisco, San Francisco, California, USA

## Abstract

**Background:**

The aim of this paper is to highlight emerging data on occupational attributable risk in asthma. Despite well documented outbreaks of disease and the recognition of numerous specific causal agents, occupational exposures previously had been relegated a fairly minor role relative to other causes of adult onset asthma. In recent years there has been a growing recognition of the potential importance of asthma induced by work-related exposures

**Methods:**

We searched Pub Med from June 1999 through December 2007. We identified six longitudinal general population-based studies; three case-control studies and eight cross-sectional analyses from seven general population-based samples. For an integrated analysis we added ten estimates prior to 1999 included in a previous review.

**Results:**

The longitudinal studies indicate that 16.3% of all adult-onset asthma is caused by occupational exposures. In an overall synthesis of all included studies the overall median PAR value was 17.6%.

**Conclusion:**

Clinicians should consider the occupational history when evaluating patients in working age who have asthma. At a societal level, these findings underscore the need for further preventive action to reduce the occupational exposures to asthma-causing agents.

## Background

In recent years there has been a growing recognition of the potential importance of asthma induced by work-related exposures, both in developed and emerging economies [1-8]. One reason for this new awareness of the importance of occupational factors is that these have been analysed using an approach based on population attributable risk (PAR). The PAR (also referred to as the population attributable fraction), can be derived from standard risk-based measures (the relative risk or odds ratio), and provides an estimate given in percent of the overall burden of disease in a population that is due to the risk factor in question [9].

In 1999, we reviewed all published reports dating back to 1966 that provided data pertinent to risk estimates for asthma in relation to occupational exposures [10]. A summary of the findings for all 31 of the reports included in this previous review, which yielded an overall PAR estimate of 15%, is presented in Table 1. A few years following that review, the American Thoracic Society (ATS) carried out a critical synthesis subsuming the salient population-based literature in this field, leading to the formal adoption of a statement, *Occupational Contribution to the Burden of Airway Disease *[11]. Overall, the PAR estimates for asthma summarized in the ATS statement also yielded a median value of 15%.

**Table 1:** Summary of previously reviewed studies where population attributable risk (PAR) for occupational exposures and asthma either have been presented or derived from published data.

Type of study	Studies Included	PAR		
		Range	Mean	Median
Cross-sectional, general population	16*	2%–45%	17%	17.5%

Case-control studies	6^†^	2%–33%	20%	21.5%

Asthma clinical cohorts or case series	6	6%–21%	12%	11.5%

Theoretical or consensus	3	2%–9%	6%	8%

All	31	2%–45%	15.2%	15%

*One study comprised of women only.

^†^One study comprised of men, only.

(summarized from Blanc and Torén, see reference 10).

Although these reviews highlighted the relative magnitude of the occupational contribution to asthma in adults, a great deal of uncertainty remained. The wide range of estimates from these various reports, (2% to 45%), even with the central tendency of 15%, further underscores their associated uncertainties. It is important, therefore, to evaluate additional relevant studies that have appeared in the interim in order to evaluate their consistency in light of previous systematic reviews. In particular, prospective longitudinal studies of risk factors in adult asthma, which were not available at the time of previous reviews, are particularly germane to the issue of occupational attributable risk. Hence, the purpose of this review is to systematically evaluate the scientific literature that has appeared since our previous review, synthesizing these new studies and integrating their findings with previously summarized data.

## Methods

### Literature Search

We identified relevant citations through three approaches. First, we carried out a systematic literature search in PubMed using the algorithm:

asthma/epidemiology/*etiology AND occup* AND adult*

We restricted this search to English language citations published from June 1999 through December 2007. There were 180 unique PubMed citations identified through this algorithm. Second, we reviewed all English-language papers identified through the Science Citation Index (SCI) as having cited our previous review [10] or the ATS statement [11] on PAR. The SCI search was limited to the same period as noted above for the PubMed search. Third, we scrutinized the reference lists from any recent review on the topic of occupational asthma, as well as the references cited in any appropriate papers identified in above, in order to identify other studies consistent with the same parameters noted previously. We restricted this review to studies published in full papers that employed general population samples or comparable samples drawn from large cohorts, such as those derived from health insurance primary care schemes. We further limited eligibility to studies that either provided an estimate of PAR for occupational exposure, provided risk estimates and exposure metrics that allowed us to calculate PAR based on published data, or, in certain cases, provided the raw incidence of occupational cases nested within total asthma incidence.

### Data Extraction and Secondary Calculations

If PAR not was provided in the published paper, we used the formula [(RR-1)/RR)*exposure frequency_cases_] to estimate PAR, where RR = the odds ratio or other risk ratio as published. In some papers, multiple estimates were provided based on differing definitions of exposure (for example, self reported occupational exposure and exposure based on a job exposure matrix [JEM]. In those cases, we also calculated the numeric average of the principal values presented. We summarized data from the identified studies calculating the mean and median as well as the range of estimates. For summary data, we categorized by the methodology used: longitudinal cohort, case-control, cross-sectional, and insurance-scheme clinical series. Where indicated, we characterized the PAR estimate as based on all asthma (regardless of age of onset) or adult asthma asthma only.

### Overall Synthesis of Studies

As a final analytic step, we carried out an overall of available studies, including both the new studies we identified for this review and studies that had been used in our previous review [10]. In this integrated analysis we only used data-based, full published papers for this synthesis, excluding abstracts, editorials, and theoretical PAR estimates. These eligibility criteria were also applied to older reports even if they had been included in previous reviews [10,11]. Second, we excluded reports based on clinical asthma series. We did, however, retain in this analysis two recent studies carried out in large U.S. Health Maintenance Organization (HMO) cohorts because these were systematic and prospective. Third, we excluded data drawn from subsets of larger studies that were reported separately when analysis of the overall data set was also available [12-15]. Fourth, we also excluded cross-sectional analyses from cohorts that were later analyzed in prospective studies [16-18]. Fifth, we also excluded papers which were based on imprecise surrogates of asthma (eg, wheezing alone). As noted above, if multiple PAR estimates were available for the same cohort, we averaged these results.

## Results

### Type and Geographic Distribution of Studies

We ultimately identified 21 publications (based on data from 18 study populations) meeting inclusion criteria. Of these, six were longitudinal general population-based studies [19-24], and four were case-control studies based on three populations [26-29]. Eight other publications (based on data from seven data sets) were cross-sectional analyses of general population based samples [30-37]. Finally, three other publications (based on two study populations) were based on analysis of incident cases from large insurance schemes characterized as closed-panel health maintenance organisations (HMOs) [38-40]. There was a wide range of different countries represented in these studies, and altogether, 19 countries on six continents are represented in the studies analyzed.

### Prospective Longitudinal General Population-based Studies

Table 2 summarizes data on PAR from the six longitudinal analyses included [19-24]. A cohort of Israeli soldiers without asthma or asthma symptoms and with normal lung function were followed for 30 months [19]. This study had been included in the data used for the ATS estimates, but not in our original review [11]. Of 34,038 soldiers in the combat units, 405 soldiers developed asthma; of 16,054 soldiers in maintenance units, 131 developed asthma. This incidence compared to 52 new cases of asthma among 8,956 soldiers with clerical tasks. The relative risk estimates for new-onset asthma was 2.0 and 1.4 for the combat and maintenance units, respectively (p < 0.05). Based on these data, the derived PAR has been estimated to be 44% [11].

**Table 2:** Description of longitudinal general population cohort studies of asthma published June 1999–2007 in which the population attributable risk (PAR) for occupational exposures and asthma was either published or can be derived.

Ref	Subject n Case n	Country	Asthma Definition	Occupational Exposure	PAR
19	59,058 588	Israel	Physician-diagnosed	Military exposures: Combat or maintenance versus clerical	44%*

20	1,852,848 49,575	Finland	Incident asthma symptoms and at least one criteria of airway reversibility	Occupations at baseline *a priori *classified as exposed	29% men, 17% women, weighted 22%

21	2,723^†^, 101	Norway	Physician-diagnosed incident asthma	Self-reported exposure to much dust or fumes at baseline	14%

22	52,325 1,426	Singapore	Adult-onset physician-diagnosed asthma	Occupations at baseline *a priori *classified as exposed to: I: dust, II:smoke, III: vapors	I – 2.7%*, II – 1.7%*, III – 4.2%*, cumulative 8.6%

23	6,837 133 3,994 38	International	A. Incident asthma symptoms or medication B. Incident asthma symptoms or medication and new hyperesponsiveness	I. Exposure to high-risk substances (at baseline and during follow-up) by job-exposure matrix II. Occupations *a priori *classified as exposed	AI – 11%, AII – 1.7%, BI – 23%, BII – 26%, mean 18.5%*

24	5,933^‡^ 271	Sweden	Physician-diagnosed asthma	Manual workers in industry	9%

* Derived from published data

† Population for analysis excluded subjects with asthma at baseline, see reference 25

‡ The actual study population was less after excluding those with baseline asthma

A study of three temporal cohorts of employed Finns (initiated 1985, 1990 and 1995; aged 25–59 years at each baseline) followed each group for five years each (thus without overlap) [20]. This cohort overlaps, in part, with that in an earlier paper from Finland [10,17]. The onset of asthma during follow-up was determined through the Finnish National Register for Reimbursement of Asthma Medication. For the patient to qualify for reimbursement, a physician must certify a valid diagnosis of asthma, including objective documentation of variable airway obstruction. Those with asthma at baseline of each 5-year time cohort were excluded, allowing for identification of incident asthma cases. Exposure was defined *a priori *on the basis of occupational titles dichotomized to administrative (referent) vs. non-administrative jobs. There were 20,777 and 28,798 incident cases of asthma among men and women, respectively. The exposure prevalence (occupations classified as exposed) among the cases was high, 94% (19,502/20,777) among the men and 82% (23,563/28,798) among the women. The incidence rate ratios of asthma were estimated comparing non-administrative work with administrative work resulting in relative risk of 1.45 in men and 1.27 in women. The incident asthma PAR associated with occupational exposure (non-administrative work) reported by the authors was 29% (95% Confidence Interval [CI] 25–33%) for men and 17% (95% CI 15–19%) for women, yielding a gender-weighted overall PAR value of 22%.

A Norwegian study consisted of follow-up of a general population sample of 3,886 subjects [21]. The baseline cross-sectional analysis of this cohort was included in the previous reviews of PAR [10,16]. The age range at baseline was 15 to 70 years. At ten year follow-up (1996), approximately 2,819 subjects, asthma-free at baseline, were successfully re-examined. Incident asthma was defined as an affirmative answer to "having been hospitalized or treated by a physician for asthma." The occupational exposure was defined by self-report to dust or fumes. The exposure prevalence was 28%, with a considerable difference between men (44%) and women (13%). Based on the data presented, the exposure prevalence among the subjects with asthma was 39%. The relative odds (OR) estimate for incident asthma in relation to being ever-exposed to dust or fumes was 1.6 (95% CI 1.01–2.5) using adjusted logistic regression models. The dust and fume-associated PAR for incident asthma reported by the authors was 14% (95% CI -1.2 – 27.6). Of note, although the OR was statistically significant, the CI for the PAR estimate did not exclude the zero value.

In a study of the ethnic Chinese population in Singapore 63,257 randomly selected subjects completed a questionnaire about comprising 45 items about occupation and a medical history [22]. Their age at baseline was 45 – 75 years. After about 6 years, 52,325 subjects (83%) answered a follow-up questionnaire including items about physician-diagnosed asthma with onset of symptoms after 18 years of age. Subjects with childhood asthma were excluded, but other subjects with asthma at baseline were not. Thus, this study design can be best described as a longitudinal study with prevalent cases of adult asthma. The ORs for adult-onset asthma in relation to baseline occupational exposures based on job categories were: 1.14 (95% CI 1.90–1.30) for dust, 1.34 (95% CI 1.15–1.56) for vapors, and 1.13 (95% CI 0.97–1.33) for smoke. Based on occupations, dust exposure assigned to 22% of the cases, vapour to 17% and smoke to 14%. The derived PAR estimates are shown in Table 2. Although data on overlapping exposures were not provided, the method of job-based assignment appeared to generate mutually exclusive categorizations, although this was not explicit in the methods as described. Moreover, the ORs were calculated such than anyone with concomitant exposure would have been included in the referent (non-exposed group), biasing the estimated risks toward the null. Thus, it is reasonably conservative to add together these PAR estimates, yielding an overall value of 8.6%.

The European Respiratory Health Survey (ECRHS) began as an international cross-sectional study from 28 centers in 13 countries. An analysis of cross-sectional data from the ECRHS I yielded a PAR estimate of 9% for occupation and prevalent asthma [10,18]. Approximately ten years after baseline, an international follow-up was carried out (ECRHS II). Subjects with asthma, wheezing and dyspnoea at baseline were excluded. Incident asthma was defined in two principal ways: first, based on a reported asthma attack or use of asthma medication in the 12 months preceding the interview, and second, a more restrictive case definition also requiring a positive methacholine challenge test [23]. Exposure in ECRHS II was also assessed by two methods, one using a broad at-risk occupational classification and the second linking the occupations to an asthma-specific JEM comprising 18 substances *a priori *classified as carrying high risk for asthma. The OR for asthma defined by symptoms or medication was 1.6 (95% CI 1–1 – 2.3) and for the latter in combination with a positive metacholine challenge test, based on a smaller sample size with available test data, 2.4 (95% CI 1.3–4.6). The associated PAR estimates are shown in Table 2. The average PAR derived from these estimates is 18.5%.

A study on a random general population sample from northern Sweden comprised 6,837 subjects aged 35 to 75 yrs at baseline which were followed-up after 10 years with a respiratory questionnaire [24]. Subjects with physician-diagnosed asthma at baseline were excluded. The risk for incident asthma in among manual workers in industry was increased (OR = 1.7, 95% CI 1.0–2.7); the reported PAR was 9% (95% CI 0–14%).

### Case-Control Studies

As shown in Table 3, four papers reporting data from three case-control studies of asthma and occupation met inclusion criteria [26-29]. In a Swedish study, prevalent cases of asthma among persons 20–65 years of age within a defined geographic area were identified as all subjects seeking medical care for asthma during a period of 18 months, using computerized data from multiple regional sources [26] The diagnosis was based on a combination of symptoms and objective signs of reversible airway obstruction. There were 120 cases of adult-onset asthma included in the analysis, along with 446 referents randomly selected from the general population. Occupational exposures (based on a JEM and by self-report) before the onset of asthma (and a corresponding anchor year for the controls) were considered. Among the asthma cases, occupational exposure (dust, fume or vapors) prevalence was 52% as assessed by job-exposure matrix and 43% based on self-report. Exposure was associated with asthma by both measures: by JEM, OR = 1.5 (95% CI 1.0–3.3); by self-report, OR = 2.5 (95% CI 1.5–3.9). The estimated PAR values that can be derived from these data are 17.2% and 25.5%, respectively, with an average value of 21.4%.

**Table 3:** Description of case-control studies published June 1999–2007 where population attributable risk (PAR) for occupational exposures and asthma was either published or could be derived.

Ref	Cases	Controls	Country	Asthma definition	Occupational exposure	PAR
26	120	446	Sweden	Adult onset asthma based on diagnosis in medical records	I. Occupations classified as exposed.	I. 25.5%*
					II. Self-reported exposure	II. 17.2%*
						Mean 21.4%*

27	I. 172	I. 285	France	I. Asthma, any age onset	Occupations at risk, defined *a priori *by a job-exposure matrix	I. 10.0%*
28	II. 48	II. 228		II. Adult-onset, severe		II. 29.7%*
	III. 43			III. Adult-onset, mild		III. 2.7%*^†^
				II.+III. All adult onset asthma		II+III. 16.9%*
						Mean 13.5%*

29	373	4,329	Australia	Adult-onset physician-diagnosed asthma	Occupations or exposures *a priori *classified as having an increased risk	9.5%‡

*. PAR% derived from published data

† Exposure was weakly and non-significantly associated with mild adult-onset asthma, OR = 1.2, 95% CI 0.5–3.0.

‡ A statistically significant, but low prevalence of acute irritant exposures accounted for an additional PAR of 0.2%

A second case-control investigation, a French asthma genetics study, used a job-exposure matrix to classify exposure in two separate analyses of the data set [27,28]. In the first, 172 ever-employed asthma cases (adults with asthma since childhood and adult-onset, combined) and 285 controls were analyzed [27]. For cases, exposure by the JEM was based on the job held at the time of asthma, while for childhood-onset asthma the assignment was based on current job. Exposure based on the JEM classification was associated with risk of asthma (OR 1.7; 95% CI 1.1–2.7); the associated PAR (derived from these data) is 10%. A further analysis of this case-control data set re-applied the same job-exposure matrix (based on current job), but analyzed severe and mild adult-onset asthma separately [27]. In this analysis, 19 (40%) of 48 severe cases were exposed compared to 34 (15%) of 228 controls (OR 4.0; 95% CI 2.0–8.1). The associated PAR (derived from these data) is 29.7%. The OR for mild adult-onset asthma was minimally elevated (OR 1.2; 95% CI 0.5–3.0) and dilutes the overall PAR% of mild and sever asthma combined to 16.9%. The overall average of the two reports is 13.5%.

In a third case-control study, from Australia, a random population sample of 5,331 subjects aged 18–49 yrs completed a questionnaire comprising items about physician-diagnosed asthma and occupational exposures, with a somewhat restricted study n = 4,366 used in the key analysis [29]. For the analysis of occupational risk, cases were defined as persons reporting adult-onset asthma; referent subjects never reported asthma, asthma medications, or asthma symptoms. Only occupational exposures (34% among cases) before the onset of asthma (and a corresponding anchor year for the controls) were considered. Exposure to any high risk exposure was associated with adult-onset asthma (OR = 1.51, 95% CI 1.19–1.92). The reported PAR for *a priori *high-risk jobs or reported high risk exposures was 9.5%.

### Cross-sectional Studies

Eight reports of cross-sectional analyses met study inclusion criteria (Table 4) [30-37]. A random population survey was carried out in six Canadian communities following the ECHRS baseline protocol [30]. These data, however, were not included in the baseline ECHRS cross-sectional analysis [18] and no follow-up has taken place. Exposure was defined as work in a high risk occupation-industry (*a priori*) or report of a specific occupation before or at the time of adult onset asthma. Among 2,974 subjects analyzed, 166 had adult-onset asthma. Preceding exposure was associated with asthma (OR 1.48; 95% CI 1.05–2.09). The estimated PAR, as reported, was 18.2%.

**Table 4:** Cross-sectional general population studies published June 1999–2007 where the population attributable risk (PAR) for occupational exposures and asthma was either published or derived.

Ref	Subject n Case n	Country	Asthma definition	Occupational exposure	PAR
30	2,974 166	Canada	Adult-onset physician-diagnosed	*A priori *high risk occupations or a report of exposure before onset of asthma	18.2%

31	14,151 976 (ever asthma) 13,445 270 (asthma, current job)	France	A. Ever asthma attack or dyspnea with wheezing; B. Adult-onset during or after current job	I. Self-reported exposure to gases, dusts and fumes II. Job-exposure matrix (excluding jobs with imprecise estimates, n = 10,560)	A., I. 9% A., II 1%* B., I. 14% B., II. 7% Mean 7.8%^†^

32, 33	5,022 185	US	Physician-diagnosed, ever	I. Occupations *a priori *classified at-risk II. Industries *a priori *classified at-risk	I. 26.0% II. 36.5% Mean 31.3%^†^

34	1,482 77	U.S.	Physician diagnosed, adult-onset	I. Self-reported exposure, vapors, gas, dust or fume II. Job-exposure matrix III. Both I and II	I. 17% II. 5%^‡^ III. 14%^‡^ Mean 12%^†^

35	I.16,646 1,471 II. 11,337 641	U.S. (three states) U.S. (two states)	Both use self-reported, health professional- diagnosed adult-onset asthma	Told by a health care provider that asthma was work-related	I.6.0%^† ^II.8.1%^†^ Mean 7.0%^†^

36	1,922 227	Brazil	Bronchial hyperresponsiveness and adult-onset asthma symptoms	Self-reported exposure, vapors, gas, fumes or humidity	22.9%^†^

37	13,826 523	South Africa	Physician diagnosed asthma, ever	Ever regularly exposed to smoke, dust, fumes or strong smells or worked underground in a mine	13.6%

*The PAR% for ever asthma was reported for the entire group and is included here as a conservative value. †PAR% derived from published data

In a French study, 14,151 subjects were investigated in 1975 with a questionnaire regarding self-reported asthma, occupations and self-reported exposure to dusts, gases and fumes [31]. The exposure in the current or most recent job was assessed either by self-report or by a job-exposure matrix. For the latter, a number of subjects with imprecise job-exposure estimates were excluded form a final analysis. It is also noteworthy that households headed by manual workers were excluded from the cohort at inception. This suggests that PAR estimates from this cohort are likely to be conservative given that those most likely to be exposed were not studied. The different reported PAR values are presented in Table 4. This average value we calculated based on these was 7.8%.

Arif and co-workers have published two cross-sectional analyses of the NHANES III data drawn from a national U.S. weighted randomized sample [32,33]. In the first publication, they analysed the risk for work-related asthma based on industry of employment considered *a priori *to carry increased risk [32]. The exposure prevalence among the 185 cases of asthma was high (89%), with an associated PAR estimate reported to be 36.5%. In a second analysis, the risk for work-related asthma was based on occupation (also categorized for risk on an *a priori *basis). The exposure prevalence among the same 185 cases of asthma was also high (68%), with an estimated PAR reported to be 26%. The averaged value of the two estimates we derived is 31.3%.

A U.S. random-digit-dial survey used data from 1484 older adults (aged 55–75 years, of whom 77 reported a physician's diagnosis of adult-onset asthma) to estimate the PAR for asthma comparing a JEM to self-report of vapors, gas dust and fume [34]. The analysis used the longest held job without regard to age during adulthood in regard to employment; subjects with chronic obstructive pulmonary disease or childhood onset asthma were excluded. Asthma was associated with occupation based on self-reported exposure (OR 1.7; 95% CI 1.03–2.8), but only weakly by JEM (OR 1.2; 95% CI 0.7–21). The PAR was 17% (95% CI 0–32%) and 5% (95% CI -11–19%), respectively. The average of these two reported values is 11%.

In another cross-sectional study from the U.S., adults who were identified by a random-digit-dial in three states completed a telephone survey [35]. The prevalence of work-related asthma was defined by one measure asking if the subjects with physician-diagnosed asthma were ever told by a health care provider that their asthma was work-related or whether they had ever told a provider this was the case. Although California was also surveyed, only for Massachusetts (approximately n = 449) and Michigan (approximately n = 193) were data specific for adult-onset asthma available. For these two samples, 8.1% and 6.0% had work-related asthma by this definition, respectively. This yields a sample size-weighted average value of 7.0%.

In a cross-sectional analysis of a general population-based sample from Brazil, subjects with a positive bronchial methacholine challenge test who also reported a temporal association between asthmatic symptoms and work were compared with referents with a negative bronchial methacholine challenge test [36]. The PAR of asthma related to self-reported exposure to dusts, gases, fumes, vapors, chemical products, paints and humidity is 23%, based on this raw incidence value. Based on the design of this study, there was likely to be inclusion of subjects with work-aggravated asthma, even though the authors did classify pre-existing childhood-onset asthma separately. Hence the PAR may overestimate asthma attributable to work etiologically. Nonetheless, the study is important because the paucity of such data from countries with emerging economies.

Data from a South-African national health survey were used to estimate the risk for ever physician-reported asthma in relation to "ever worked in a job regularly exposed to smoke, dust, fumes or strong smells or ever worked underground in a mine" [37]. The PAR reported from that study was 13.6%.

### Asthma Incidence in Health Maintenance Organization (HMO) Populations

In addition to the data in Table 4, two relevant analyses have been published based on incident cases of asthma identified through prospective study of HMO cohorts. In the baseline populations, asthmatic subjects (based on diagnosis and medication use) were excluded. Each new case of asthma was classified based on asthmagenic workplace exposures and whether symptoms were work-related. In one of these studies, 1747 potential incident asthma cases were identified, of which 352 were interviewed and confirmed [38]. Ultimately, 33% of these were classified as work-related. In another study based in an entirely different U.S. HMO but employing comparable methods, 24% of all 405 incident or "reactivated" cases of asthma were attributed to occupational exposures [39]. Interim data from the same group found that only 5% of incident cases were diagnosed by a treating clinician as occupational in aetiology, but since only 7% were documented to have been asked about work, the 5% value is likely to be an overly conservative underestimation of the PAR [40].

### Data Synthesis

Table 5 presents a synthesis of available studies from the current analysis and from our previous review. As shown in Table 5, there were six longitudinal studies included, and, based on these studies, 16.3% (median) of all asthma, adult-onset by the nature of these studies, is attributable to occupational exposures. Table 5 also includes six PAR estimates derived from case-control studies; three of these estimates (two based on means of more than one estimate) were also included in Table 3 and three others were included in both our previous review and in the ATS statement [41-43]. Three case-control studies from our previous review have not been included, as they were either published only as abstracts or only use a case definition comprising wheeze only. Taken together, the six studies yield a median PAR estimate of 12.2%. A PAR estimate (median) 17.6% is obtained from the 14 cross-sectional studies included in Table 5[31-37,44-51]. Few studies have separated the analysis with regard to gender. We identified five papers with separate estimates for males [17-20,43] and females [17,18,20,43,45]. The resulting median estimates are 9.1% for males and 11.5% for females.

**Table 5:** Synthesis of previously and currently reviewed studies regarding population attributable fraction (PAR) for occupational exposures and asthma.

Type of study	Studies Included	Ref	Range	Mean	Median
Current review					

Longitudinal	6	19–24	8.6%–44%	19.3%	16.3%

Case-control	3	26–29	9.5%–21.4%	14.8%	13.5%

Cross-sectional	7	31–37	7.0%–31.3%	16.1%	13.6%

Current and earlier review					

Longitudinal	6	19–24	8.6%–44.0%	19.3%	16.3%

Case-control	6	26–29, 41–43	9.5%–36.0%	20.7%	12.2%

Cross-sectional	14	31–37, 44–51	7%–51%	21.2%	17.6%

All	26	See above	7%–51%	20.7%	17.6%

All, adult-onset asthma only	17	19–24, 26, 28–31, 34–36, 42, 43, 45	8.6%–44.0%	18.8.5%	16.9%

Altogether, the median PAR value among all 26 studies included in Table 5 is 17.6%. We also made a separate estimation limited to analyses based on 17 values for adult-onset asthma only; this yielded a median PAR estimate of 16.9%.

## Discussion and conclusion

Since our last systematic review [10] and the ATS statement that followed it [11], valuable new data relevant to the population burden of occupation in adult asthma have appeared. In particular, multiple new analyses based on large general population-based asthma incidence studies allow for more reliable estimates of PAR. These studies collectively yield a median value of 16.3%, quite close to the findings in previous reviews [10,11]. Expanding the pool of studies to include a heterogeneous array of case-control and cross-sectional studies still results in an overall PAR estimate only modest different (17.6%), while limiting this to adult-onset asthma only (which includes all of the longitudinal analyses above, but also substantially increases the study pool) yields and estimate of 16.9%.

We excluded from this review occupational asthma incidence studies derived from surveillance data, given that sources markedly underestimate the proportion of cases attributable to work-related factors. Recent data from Finland indicate that, even after excluding officially recognized occupational asthma cases, excess risk of disease is still evident on epidemiologic grounds [52]. The remaining risk is consistent with under-detection of one half to two-thirds of cases proportionally, even for well recognized risk groups such as bakers, fur workers, and painters. In contrast to under-reporting through surveillance, there is likely to be a bias toward over-attribution in clinical case series in which the PAR is derived from the ratio "probable" occupational cases with a denominator of all asthma cases identified in a registry, clinic, or hospital data base. In particular, case series that assign a case definition of occupational asthma solely because disease has occurred in a high risk job may overestimate the proportion of all cases that are work-related. We addressed this problem by excluding such case series in the integrated estimates shown in Table 5, although we acknowledge that case series with rigorous diagnostic criteria do indeed provide useful insights [53].

We have not weighted our estimates taking into account the size of the study populations reported. We have, however, provided the number of asthma cases in each study type (Table 5); these data indicate that the longitudinal general population studies have accounted for a major part (more than nine in ten) of the asthma cases upon which the PAR estimates have been based.

There are remarkably few studies, only five, presenting separate estimates for males and females. The median estimates with regard to gender were quite similar, but there is clearly a need for more studies that stratify by gender as well as other potential covariates that may be of interest, such as smoking and atopy. We have taken a simplistic approach regarding exposure classification consistent with a PAR approach. Thus we have not analysed the impact of more specific exposures such as flour dust or diisocyanates, nor have synthesized data from industry or occupation-specific studies that may be relevant to exposure-specific risk estimates.

There have been other recent reports that were not included in our analysis that may be relevant to broader questions of occupational factors in asthma across working groups. For example, a population-based case-control study from Finland provides insights on various occupational groups associated with increased asthma risk, but does not allow for combined risk estimates from which a PAR estimate can be derived [54]. There have also been a number of studies addressing the relative frequency work-aggravated asthma, a subject beyond the scope of this systematic review [55].

The analysis presented here yields an estimate of the PAR for asthma associated with work-related exposures that is quite consistent with past estimates. The range of the single estimates from each study is quite wide, but we consider a value of at least 15% and potentially as high as 20% to be the most accurate range of the likely population burden of asthma attributable to occupational exposures.

One key lesson clinicians should take from these data is that, when assessing patients of working age who have asthma, the occupational history should be carefully considered, in particular job duties held when the asthma first became manifest. In the same vein, the public health perspective should take into account the preventive implications of such findings. These data underscore the need for further actions to reduce the occupational exposure likely to lead to work-related asthma, on both the individual and population level.

## Abbreviations

ATS: American Thoracic Society; CI: Confidence interval; ECRHS: European Respiratory Health Survey; FEV_1_: Forced expiratory volume in one second; HMO: Health maintenance organization; JEM: Job-exposure matrix; PAR: population-attributable risk; RR: the odds ratio or other risk ratio; SCI: Science citation index; U.S.: United States.

## Competing interests

We declare no conflicts of interest.

## Authors' contributions

KT and PDB were together responsible for the idea, management and analysis of data and KT prepared the final draft of the manuscript.

## Pre-publication history

The pre-publication history for this paper can be accessed here:


